# The operating performance of a biotrickling filter with *Lysinibacillus fusiformis* for the removal of high-loading gaseous chlorobenzene

**DOI:** 10.1007/s10529-014-1559-5

**Published:** 2014-06-15

**Authors:** Zhao-Xia Li, Bai-Ren Yang, Jian-Xiang Jin, Yi-Chen Pu, Cheng Ding

**Affiliations:** 1 School of Chemical and Biological Engineering, Yancheng Institute of Technology, YanCheng, 224051 People’s Republic of China; 2 School of Environmental Science and Engineering, Yancheng Institute of Technology, Yinbin Road 9, YanCheng, 224051 People’s Republic of China

**Keywords:** Biotrickling filter, Biofilms, Chlorobenzene, Gaseous chlorobenzene, Gas–liquid mass transfer, Lysinibacillus fusiformis

## Abstract

Removal of gaseous chlorobenzene (CB) by a biotrickling filter (BTF) filled with modified ceramics and multi-surface hollow balls during gas–liquid mass transfer at the steady state was by microbial degradation rather than dissolution in the spray liquid or emission into the atmosphere. The BTF was flexible and resistant to the acid environment of the spray liquid, with the caveat that the spray liquid should be replaced once every 6–7 days. The BTF, loaded with *Lysinibacillus fusiformis,* performed well for purification of high-loading CB gas. The maximum CB gas inlet loading rate, 103 g m^−3^ h^−1^, CB elimination capacity, 97 g m^−3^ h^−1^, and CB removal efficiency, 97.7 %, were reached at a spray liquid flow rate of 27.6 ml min^−1^, an initial CB concentration of up to 1,300 mg m^−3^, and an empty bed retention time of more than 45 s.

**Electronic supplementary material:**

The online version of this article (doi:10.1007/s10529-014-1559-5) contains supplementary material, which is available to authorized users.

## Introduction

Prolonged exposure to chlorobenzene (CB) contamination has mutagenic, teratogenic and carcinogenic effects on human health (Field and Sierra-Alvarez 2008). Therefore, the control and treatment of CB pollutants is important. The conventional treatment methods for CB pollutants include physical, chemical and biological processes. Physical and chemical processes, such as adsorption (Liu et al. 2011; Zhao et al. 2001), condensation (Huang et al. 2013), and photolytic degradation (Zhang and Anderson 2013), etc., often require strict operating conditions, a certain dose of other chemicals, and complex response configurations. Therefore, biological methods have become the focus of research efforts due to their low cost, simple operation, and low secondary pollution. Compared with biofilters and bioscrubbers, biotrickling filters (BTFs) offer flexible control of the spray liquid, pH, and intermediate toxic products, and have obvious advantages in the degradation of inorganic waste gases, such as ammonia (Lopez et al. 2013; Xue et al. 2010) or a variety of volatile organic compounds (VOCs) (Nicolella et al. 2009; Lebrero et al. 2012). However, the application of a BTF to CB containing waste gases has been rarely reported and basic data related to this process is lacking.

Microorganisms are the key factors that determine whether BTF systems are running well (Yang et al. 2010). The common microbial agents used in the BTF include: a single dominant species, mixed species, or decomposer communities of single- and mixed-species. Under the different process conditions, each type of agent displays different degradation efficiencies. At present, the research into the predominant strains that degrade VOCs gives preference to artificially domesticated strains and mostly focuses on bacteria.

The choice of packing materials in the BTF is also crucial (Liu and Wang 2012). Packing materials with high robustness, high porosity, large specific surface area, good hydrophilicity, high surface roughness, and moderate grain size are the most suitable for microbial attachment and gas–liquid mass transfer, and have advantages in resisting any drop in pressure.

In this report, one dominant high concentration CB-degrading strain, *Lysinibacillus fusiformis* LW13 (Li et al. 2013), was activated and cultured for amplification. It was then used to form biofilms on the packing materials in the BTF. During the stable operation of the BTF, the accumulation of intermediates and pH changes in the spray fluid were monitored and analyzed. The CB gas outlet concentration (*C*_out_), the CB gas inlet loading rate (*ILR*), the CB elimination capacity (*EC*), and the CB removal efficiency (*RE*) were evaluated while varying the spray liquid flow rate (*v*), the CB gas inlet concentration (*C*_in_), the CB gas inlet flow rate (*Q*), and the empty bed retention time (EBRT). These results will provide a starting point for future in-depth studies, and industrial applications, of CB waste gas removal by a single dominant species in the BTF.

## Materials and methods

### Materials

The packing material of the BTF was a mixture of modified ceramics, 1.2 cm × 1.5 cm, and multi-faceted hollow balls, diam. 1.5 cm. The two packing materials were inert to chlorobenzene (CB) absorption and were randomly mixed at a ratio of modified ceramics: multi-surface hollow balls = 1–1.5:1. The inoculated strain, *Lysinibacillus fusiformis* LW13 (GenBank accession number JN166076), was pre-screened with a high concentration of CB, and its ability to use CB as its sole carbon source was preserved in our laboratory. The sterilized mineral medium without the carbon source (Li et al. 2013) was used as the spray liquid and prepared before use.

### Schematic diagram and operation of the BTF

**Fig. 1:**
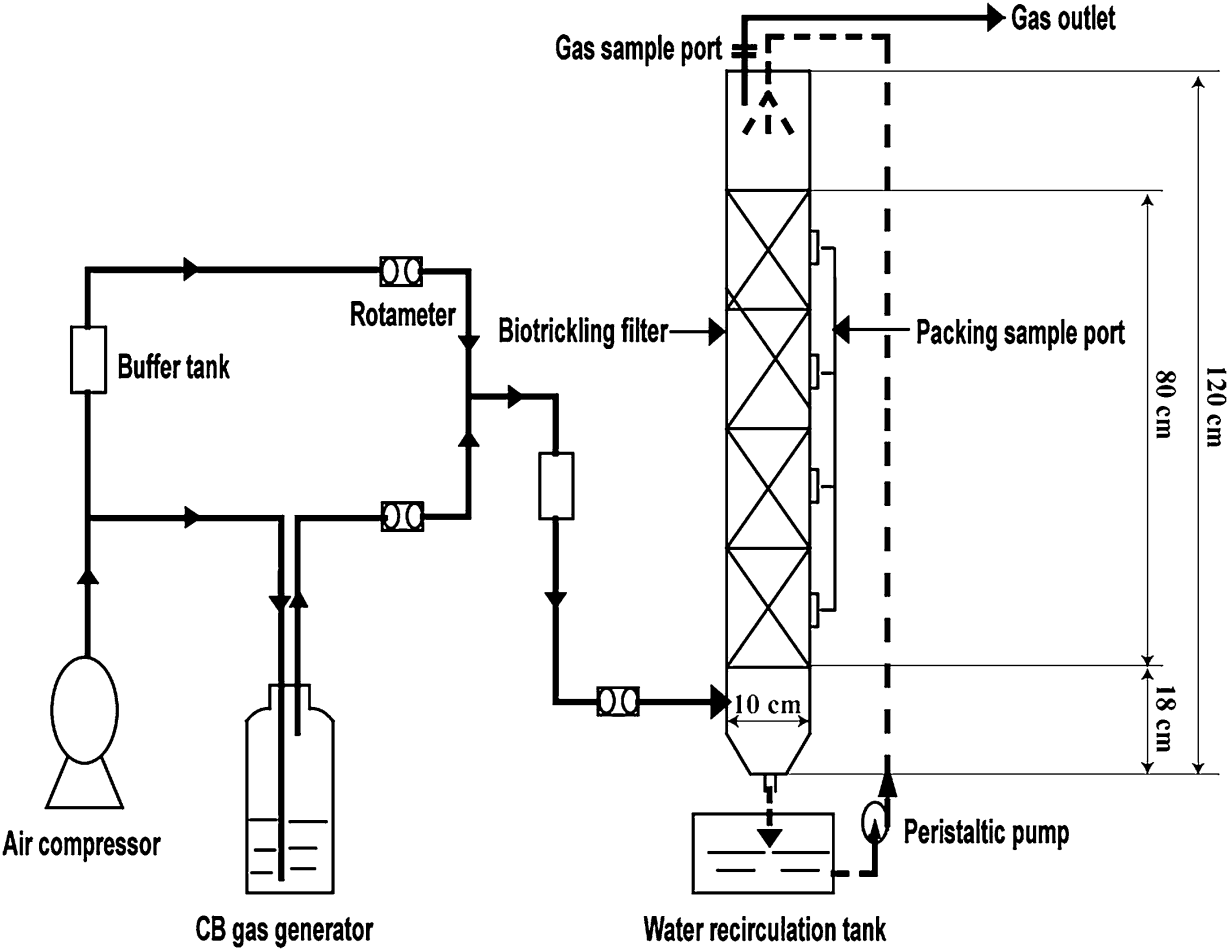
Schematic diagram of the biotrickling filter (BTF)

The BTF was made of Plexiglass, diam. 10; 120 cm (Fig. 1). The packing layer (total ht 80 cm; total volume 6.28 l) was divided by five porous clapboards into four semi-continuous separate units. The diam. of each pore was 0.8 cm and the pores were uniformly aligned on each clapboard at intervals of 0.4 cm. A rotation axis was inserted through the clapboards at the center and rotated at 40 rpm to ensure good gas–liquid mass transfer. The spray liquid, sealed in a water recirculation tank, was pumped through a peristaltic pump to the top of the BTF in a countercurrent operation and was then evenly sprayed through a sprinkler on the surface of the packing materials. The liquid CB was sealed to prevent evaporative losses to the atmosphere and boiled in a water-bath along with driving of an air flow from the air compressor to form CB gas, which flowed through a rotometer and mixed with another air flow from the air compressor to obtain the simulated waste gas. The CB loading could be controlled over an appropriate range by varying the ratio of the gas flow rate of the two rotometers. The tests were carried out under atmospheric pressure at 25 ± 2 °C. *Q* was 0.25–0.6 m^3^ h^−1^, *C*_in_ was 277–1,670 mg m^−3^, and *v* was 7.88–47.4 ml min^−1^, the corresponding EBRT was 37–90 s, and *ILR* was 15.7–146.18 g m^−3^ h^−1^. The spray liquid was refreshed once every cycle period during a 7 d cycle.

### Determination of the CB concentration

The CB concentration was determined using headspace GC. Three parallel determinations were made for each sample and the average value was used. The peak area of the samples’ standard curve was a linear function of the CB concentration. An Elite-5 capillary column (30 m × 0.32 mm × 0.5 μm) was used. The volume of the splitless injection was 500 μl, and it was injected into the vaporization chamber at 200 °C. The column flow was 1.5 ml N_2_ min^−1^ and the column temperature program was: 70 °C for 1 min, an increase to 110 °C at 10 °C min^−1^, and a hold at 110 °C for 1 min. The detector (FID) was at 250 °C.

### Determination of pH value and accumulation of metabolites

The pH of the spray liquid was measured with a pH meter. The accumulation of metabolites was evaluated at A_255nm_ (Seignez et al. 2002).

## Results and discussion

### pH and intermediate products in the BTF within a cycle period

**Fig. 2:**
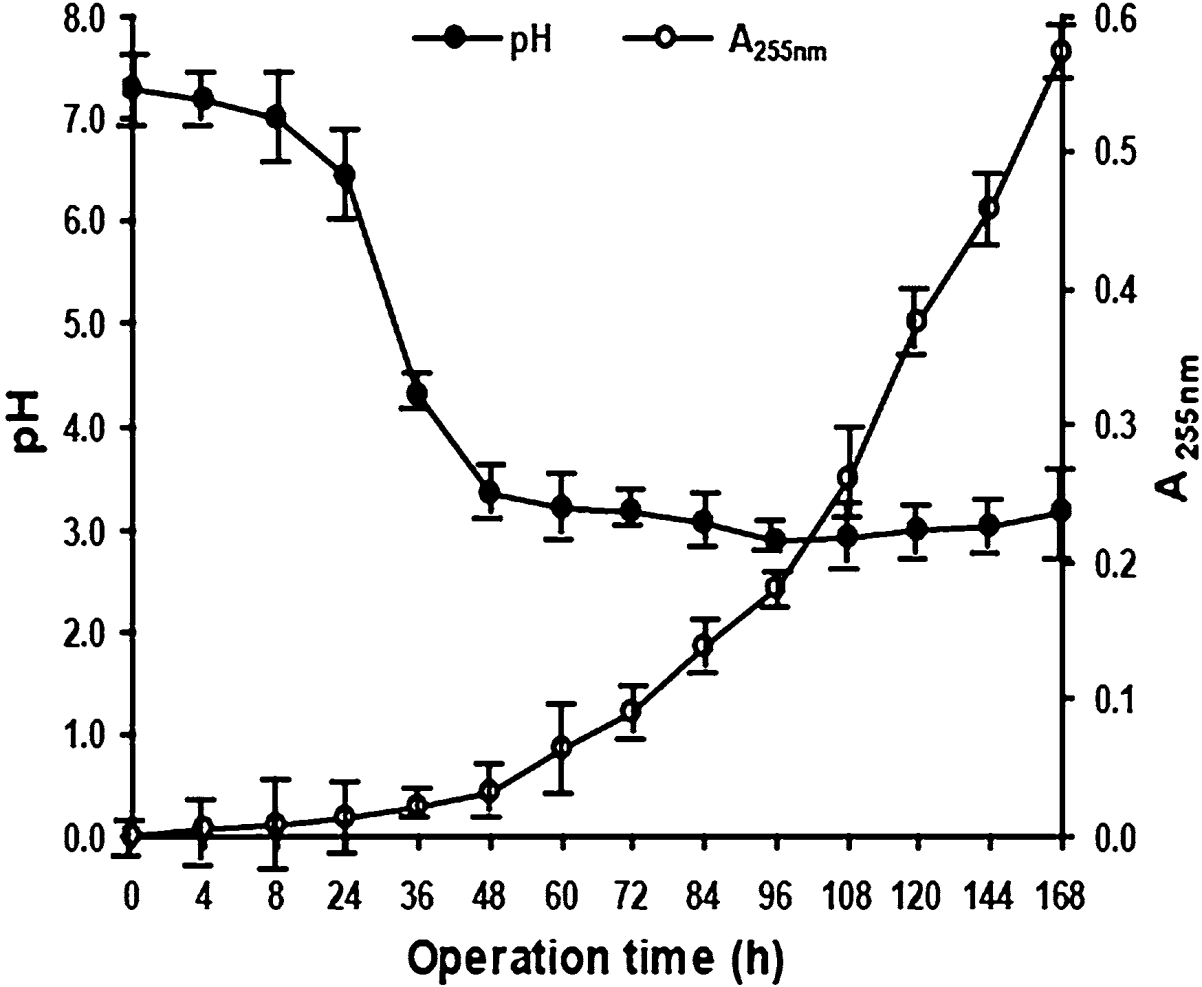
pH and A_255nm_ in the BTF within a cycle period

In the BTF, the dominant degradation strain produces HCl during the CB biodegradation process causing the system to become acidic, which can, in turn, affect the CB levels. CB biodegradation also produces a variety of metabolites, the accumulation of which can affect growth and even have a toxic effect on the dominant degradation strain. The BTF was run for one cycle under conditions of a *v* of 30 ml min^−1^, *C*_in_ of 1,200 mg m^−3^, and an EBRT of 75 s from a corresponding *Q* of 0.3 m^3^ h^−1^. To examine the effect on the CB levels as a function of pH value and the accumulation of metabolites, the pH value of the spray liquid and its A_255nm_ (Seignez et al. 2002) were monitored over time (Fig. 2).

Within 60 h, the pH of the spray liquid decreased rapidly from an initial pH of 7.3 to pH 3.2. It then fluctuated around pH 3. This demonstrated that the spray liquid became acidic and that the microorganisms in the BTF could adapt quickly and resist the acidic environment of the spray liquid. The A_255nm_ of the spray liquid could not be detected until 96 h, and then constantly increased to 0.574 at 168 h. This indicated that, as shown in Supplementary Fig. 1, the metabolites gradually accumulated as the BTF was in operation and were negatively related to the CB purifying effect. Therefore, in the CB purification process, the pH of the spray liquid should not be adjusted as an attempt to maintain neutral conditions. Rather, to maintain and maximize the utility of the nutrients in the spray liquid, it should be replaced once every 6–7 days.

### Amount of CB in the spray liquid and BTF within a cycle period

**Fig. 3:**
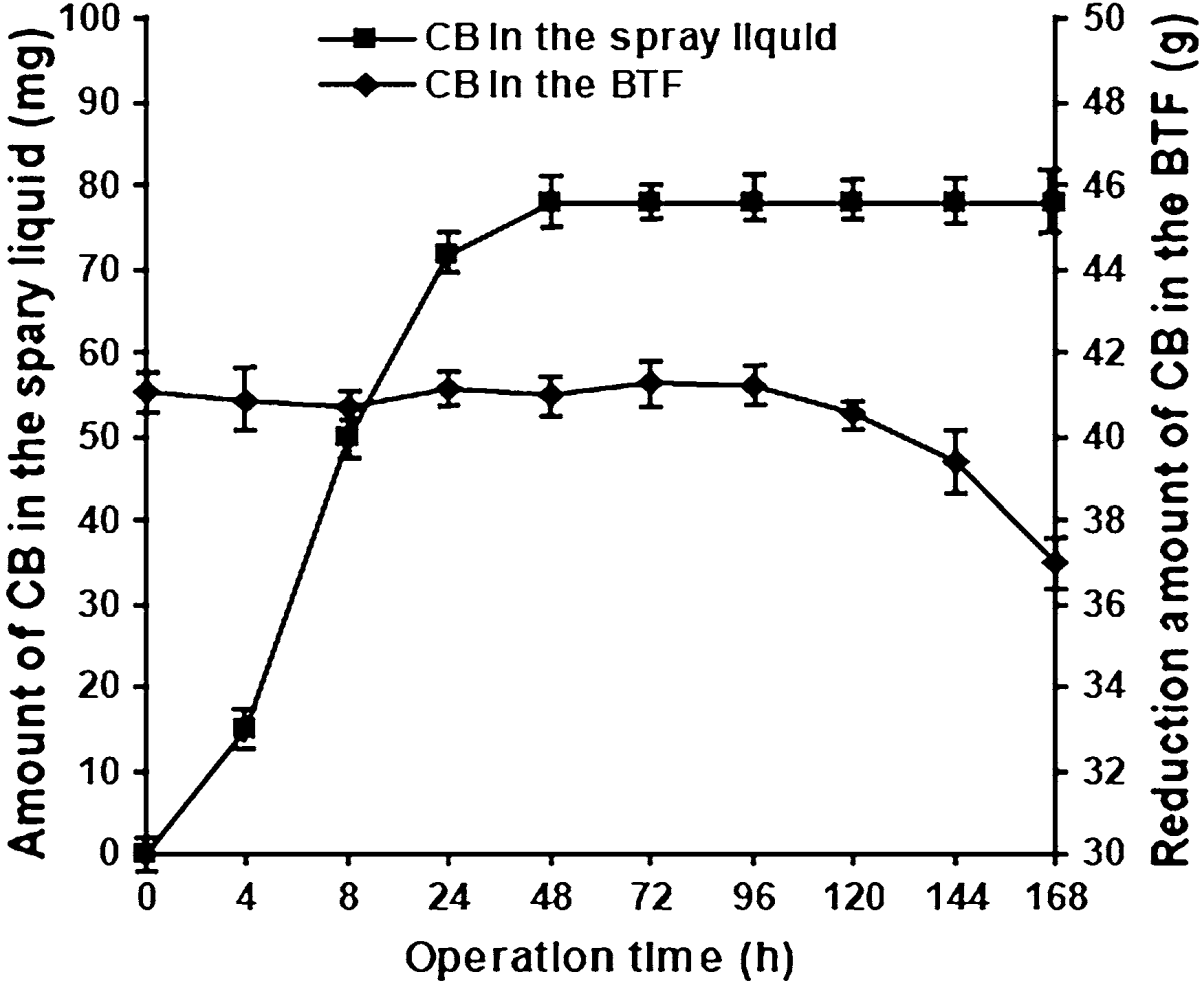
Amount of chlorobenzene in the spray liquid and the BTF within a cycle period

It is possible that a small amount of CB could be removed from the BTF by the spray liquid when the liquid is replaced. To determine this, the CB concentration in the spray liquid was continuously monitored within a cycle period, at the same time points when the pH and intermediate products in the BTF were determined. The results showed that the amount of CB in the spray liquid averaged 78 mg with only minor fluctuations after being in operation for 2 days (Fig. 3). This was well below the average cumulative reduction of CB, which was 40.4 g in the BTF in a single cycle. This illustrates that there were small levels of soluble and accumulated CB in the spray liquid. *C*_out_, shown in Supplementary Fig. 1a, indicated that the average CB loss from the gas outlet to the atmosphere was 3.2 g. Thus, the removal of CB was mostly due to the biological function of the dominant CB-degradation strain. Microstructures of the packing materials with biofilms formed by the dominant CB-degradation strain (Supplementary Fig. 2) and the CB levels (Supplementary Fig. 1) also demonstrated that the BTF system was at a steady operational state.

### CB levels as affected by C_in_

**Fig. 4:**
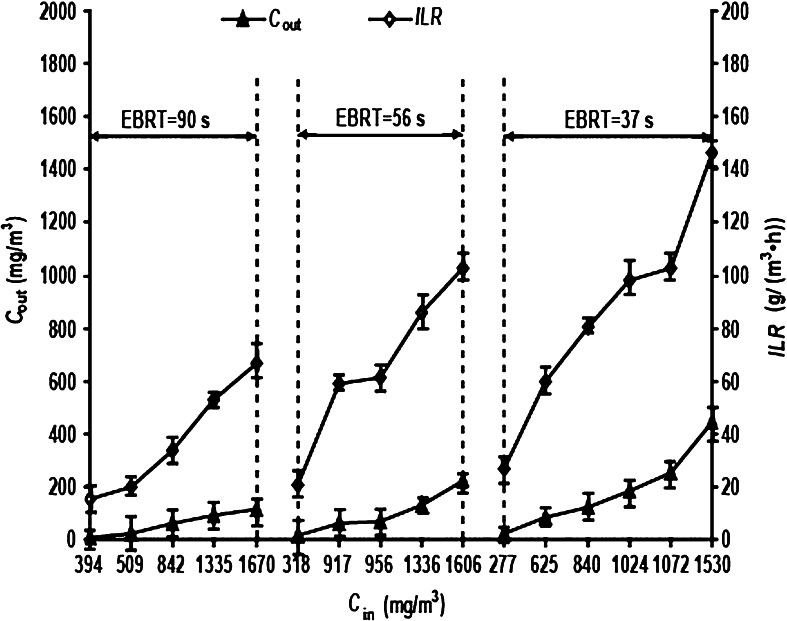
*C*_out_ and *ILR* at different *C*_in_ and EBRTs. *C*_out_ = chlorobenzene (CB) gas outlet concentration; *ILR* is the CB gas inlet loading rate; *C*_in_ is the CB gas inlet concentration; EBRT is the empty bed retention time

**Fig. 5:**
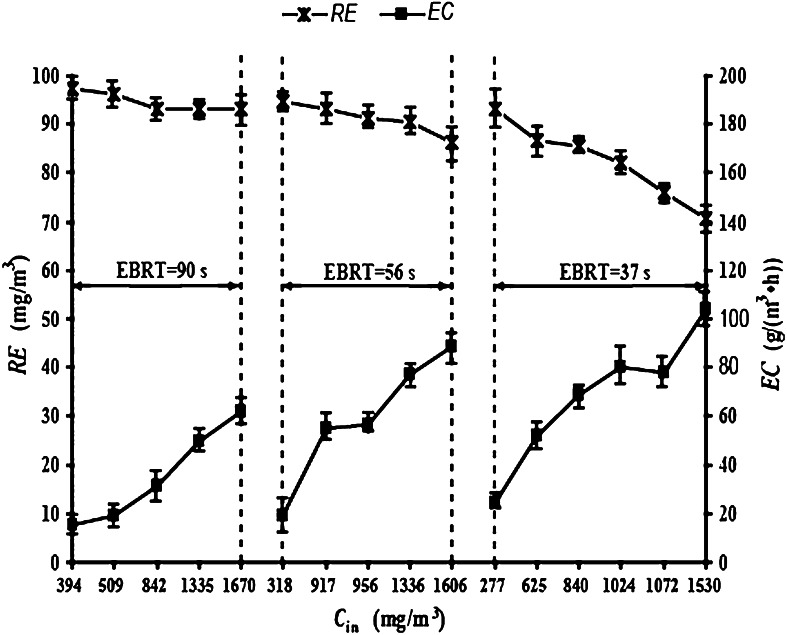
*RE* and *EC* at different *C*_in_ and EBRTs. *RE* = chlorobenzene (CB) removal efficiency; *EC* = CB elimination capacity; *C*_in_ = CB gas inlet concentration; EBRT = empty bed retention time

As CB was the sole carbon source in the BTF, *C*_in_ would play a key role in normal microbial growth and metabolism. When *v* was maintained at 30 ml min^−1^, the removal of CB was investigated at different *C*_in_ of 0.25, 0.4, and 0.6 m^3^ h^−1^ corresponding to EBRTs of 90, 56, and 37 s, respectively (Figs. 4, 5).

At a constant *v* and increasing *C*_in_, at a number of different *Q* or EBRTs, there were consistent trends in *C*_out_, *ILR*, *EC* and *RE*. When *C*_in_ was increased, *C*_out_, *ILR* and *EC* gradually increased, but *RE* decreased significantly. This indicates that *C*_in_ had a significant impact on the CB levels. Because of the reduction in the biodegradation capacity, the biodegradability of CB in the BTF decreased and the increase in *C*_out_ was larger than the change in *C*_in_. Therefore, *EC* was negatively correlated with *RE*. Thus, the CB levels could not be evaluated by simply using just *EC* or *RE*. Because of the assumption that *C*_out_ had to meet the integrated emission standard of air pollutants of China (CAIES), *C*_in_ was kept under 1,300 mg m^−3^ to improve *EC* and *RE* within this constraint.

### CB levels as affected by the EBRT (empty bed retention time)

**Fig. 6:**
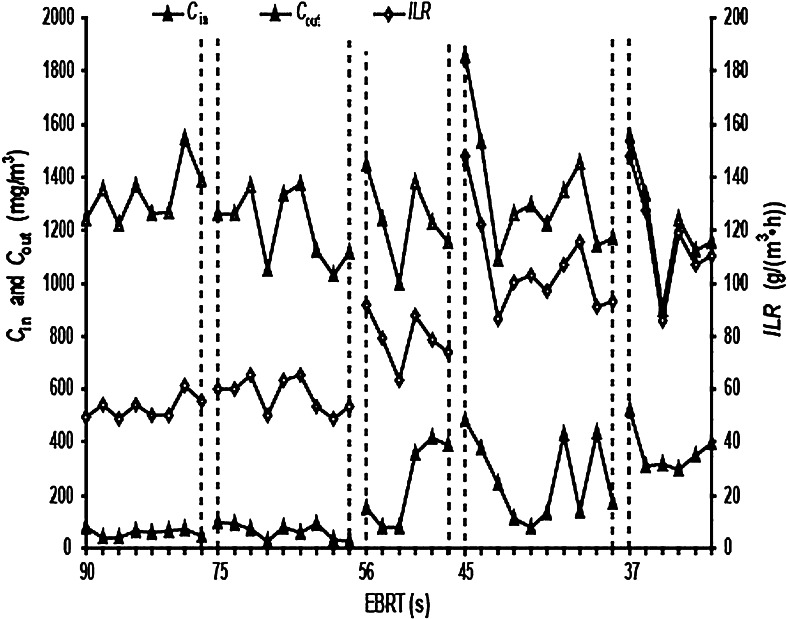
*C*_in_, *C*_out_ and *ILR* at different EBRTs. *C*_in_ = chlorobenzene (CB) gas inlet concentration; *C*_out_ = CB gas outlet concentration; *ILR* = CB gas inlet loading rate; EBRT = empty bed retention time

**Fig. 7:**
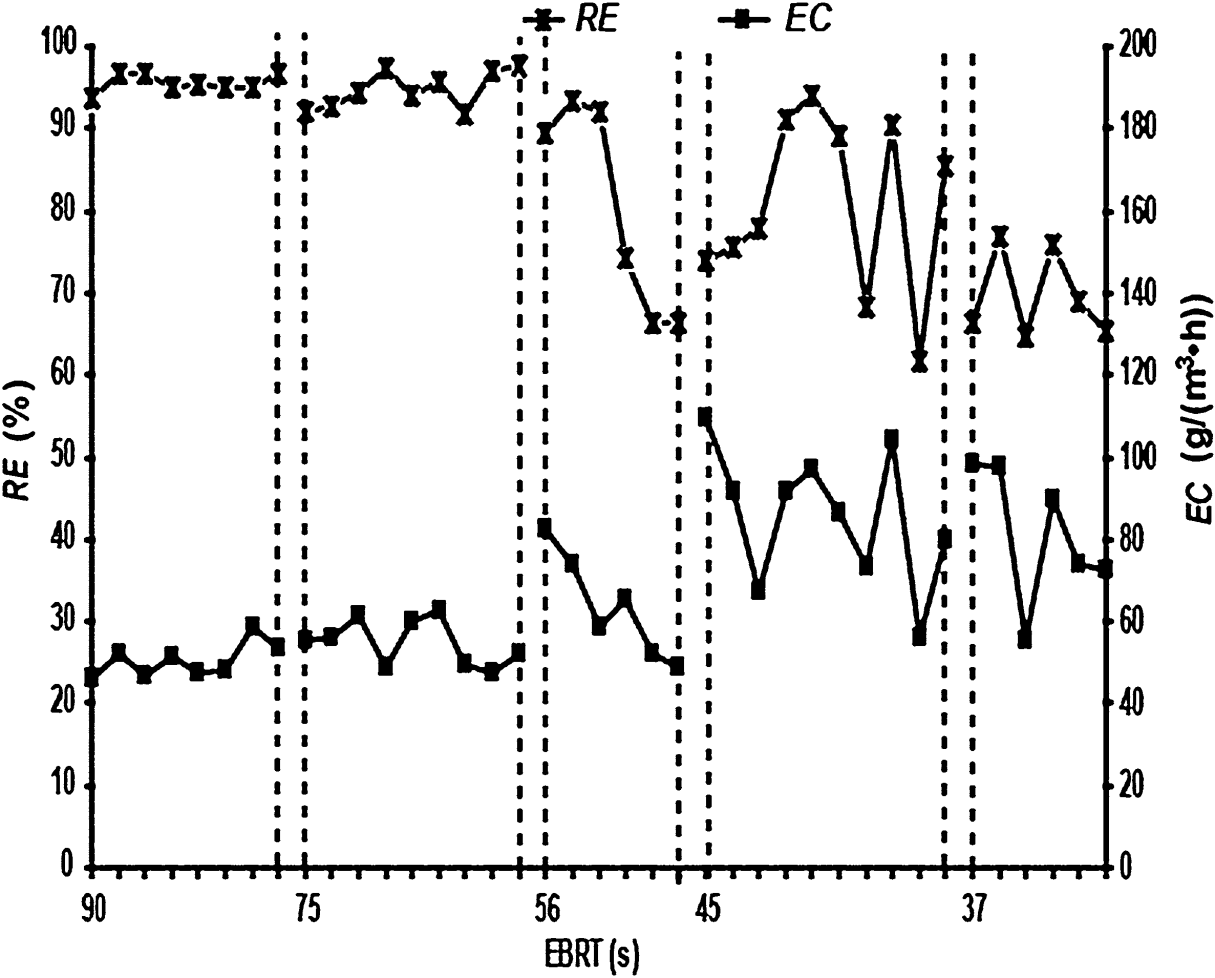
*RE* and *EC* at different EBRTs. (*RE*) = chlorobenzene (CB) removal efficiency; *EC* = CB elimination capacity; EBRT = empty bed retention time

EBRT is also an important parameter to control during the operation of the BTF. Mass transfer between the microorganisms and the packing materials is low if the EBRT is too short, and the BTF will operate inefficiently if the EBRT is too long. Given a fixed BTF volume, there is a linear, positive correlation between the EBRT and *Q*. Therefore, the CB levels as a function of EBRT were investigated at EBRTs of 90, 75, 56, 45, and 37 s (corresponding to *Q* of 0.25, 0.3, 0.4, 0.5, and 0.6 m^3^ h^−1^, respectively), at a constant *v* of 30 ml min^−1^ and a *C*_in_ of 1,250 mg m^−3^ (Figs. 6, 7).

At a constant *v*, there was a correlation between the EBRT or *Q* and *C*_in_, *C*_out_, *ILR*, *EC* and *RE*. As the EBRT was decreased by increasing *Q*, *C*_in_, *C*_out_, *ILR* and *EC* remained constant before increasing. These were all relatively large changes, although the *RE* was constant at first and then decreased. This shows that the EBRT or *Q* has a significant impact on the CB levels. At an EBRT of greater than 56 s (corresponding *Q* of < 0.4 m^3^ h^−1^), *C*_in_, *C*_out_, *ILR*,*EC* and *RE* all fluctuated over a small range. When the EBRT was <56 s (or Q exceeded 0.4 m^3^ h^−1^), *C*_in_, *C*_out_, *ILR*, *EC* and *RE* severely fluctuated over a large range, particularly at the point where the EBRT or Q was initially changed. This could be due to limitations of the test device, where changes in the EBRT could change *C*_in_. When the EBRT was further decreased, *C*_in_ was very large, so that the *ILR* of the BTF was affected by both the EBRT and *C*_in_. Therefore, to ensure that *C*_out_ meets the CAIES and to improve the operational efficiency of the BTF, the EBRT should be no <45 s from a corresponding *Q* of <0.5 m^3^ h^−1^.

### CB levels as affected by ILR

**Fig. 8:**
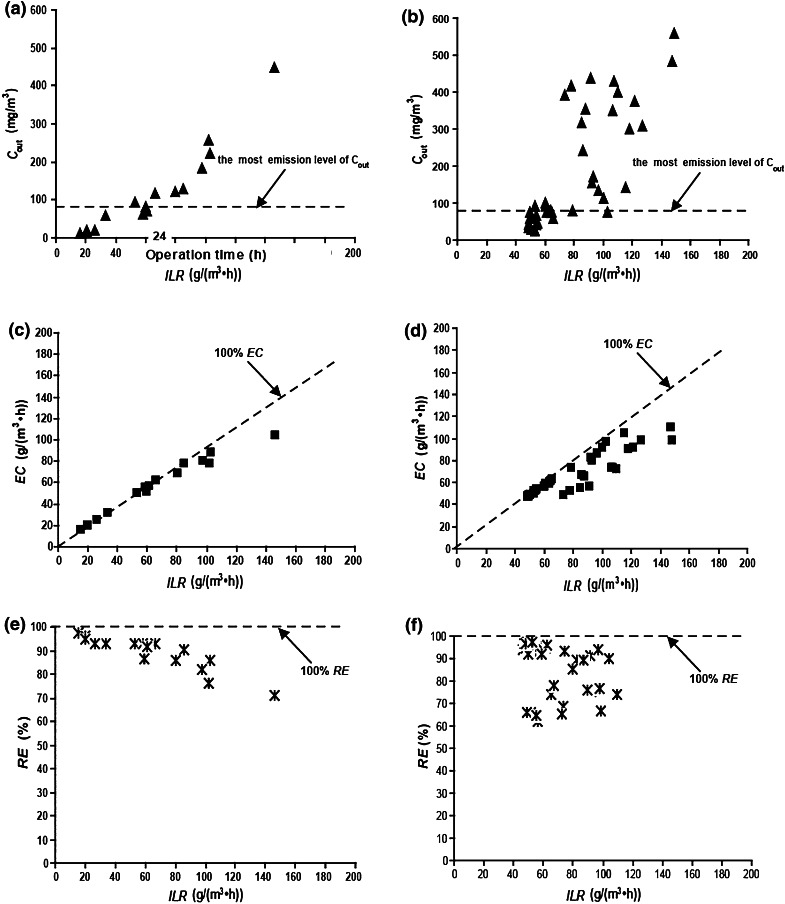
*C*_out_, *EC* and *RE* versus *ILR* **a** *C*_out_ versus *ILR* when *C*_in_ was varied. **b** *C*_out_ versus *ILR* when the EBRT was varied. **c** *EC* versus *ILR* when *C*_in_ was varied. **d** *EC* versus *ILR* when the EBRT was varied. **e** *RE* versus *ILR* when *C*_in_ was varied. **f** *RE* versus *ILR* when the EBRT was varied *C*_out_ = chlorobenzene (CB) gas outlet concentration; *EC* = CB elimination capacity; *RE* = CB removal efficiency; *ILR* = CB gas inlet loading rate

Gas purification processes are controlled by the gas–liquid mass transfer rate and the biodegradation rate. At low pollutant loads, the biodegradation rate was larger than the mass transfer rate and therefore the process was controlled by the mass transfer rate. However, at high pollutant loads, the biodegradation rate was less than the mass transfer rate, so the process was controlled by the biodegradation rate. *ILR* depends on both *Q* and *C*_in_. Therefore, a further investigation of the CB levels, as affected by *ILR*, was performed (Fig. 8).

When *ILR* was increased, *C*_out_ and *EC* increased significantly but *RE* still decreased. This means that *ILR* had a more significant impact on the CB levels in the BTF than did *v*. At low *ILR*, *C*_out_ could be maintained to meet the CAIES with a *RE* of more than 90 % and a linear, positive correlation between *EC* and *ILR*, which indicated that the mass transfer process and the biodegradation process were working well. When *ILR* was gradually increased, *C*_out_ exceeded this value, and *EC* and *RE* increasingly deviated from 100 % removal efficiency. This was probably because the mass transfer process became blocked and the biodegradation process began to play the predominant role. Thus, when the *ILR* was lower, the degradation of CB was closer to 100 %. When the *ILR* was higher, the total degradation of CB was limited by the mass transfer and biodegradation capacity of the BTF. Figure 8 also demonstrates that the distribution of *C*_out_, *EC* and *RE* at different EBRTs was more discrete and more disordered than at different *C*_in_. This indicated that the EBRT had a greater impact on *ILR* than *C*_in_, which indirectly affected the CB levels of the BTF. To ensure that *C*_out_ meets CAIES, the maximum *ILR* of the BTF can be as high as 103 g m^−3^ h^−1^ with a maximum *EC* of 97 g m^−3^ h^−1^ and a maximum *RE* of 97.7 %, by adjusting *v*, the EBRT, and *C*_in_.

In conclusion, a BTF with biofilms of the dominant degradation strain, *Lysinibacillus fusiformis* LW13, stably ran and eliminated significant amounts of CB in the BTF. This study demonstrated a particular advantage in treating high-loading gaseous CB with this setup as compared with using acclimated sludge (Zhou et al. 2011) or another single dominant species (Zhang et al. 2011). *v* (Supplementary Fig. 3), *C*_in_, the EBRT and *ILR* affected the CB levels, with the three latter factors displaying the most significant effects. The microorganisms in the BTF adapted to, and resisted, the acidic environment of the spray liquid, while the levels of metabolic intermediates could be monitored and used as a replacement signal for the spray liquid. Of the three indicators, *C*_out_, *EC* and *RE*, that reflect the BTF’s CB purification performance, *C*_out_ was the most direct and sensitive.

## Electronic supplementary material

Below is the link to the electronic supplementary material.

Chlorobenzene (CB) level within a cycle period. **a** CB gas inlet (C_in_) and outlet (C_out_) concentrations and inlet loading rate (*ILR*) within a cycle period. **b** CB removal efficiency and CB elimination capacity within a cycle period. Over one cycle of operating the BTF, the CB gas inlet concentration (C_in_) and inlet loading rate (*ILR*) were stable and fluctuated within the appropriate range. However, the CB gas outlet concentration (C_out_) initialy maintained an acceptable fluctuation and then gradually increased after 96 h and exceeded the integrated emission standard of air pollutants of China (CAIES) at 144 h Supplementary Fig. 1 (Part **a**). Nevertheless, the CB removal efficiency (*RE*) and the CB elimination capacity (*EC*) held steady in the appropriate range although they fluctuated significantly until 144 h when they rapidly decreased. Supplementary Fig. 1 (Part **b**) shows that the increase in C_out_ was the reason behind the reduction in *RE* and *EC*. Therefore, C_out_ was the most direct and sensitive indicator of CB purifying performance in the BTF. The CB purifying effect decreased after extended operation of the BTF for 6-7 d under a constant spray liquid.

Microstructure analysis of the packing materials with biofilms by ESEM. **a** ESEM of a multi-surface hollow ball with no biofilms. **b** ESEM of a multi-surface hollow ball with biofilms (× 2500). **c** ESEM of modified ceramics with no biofilms. **d** ESEM of modified ceramics with biofilms. Scale marker bars = 20 µm in all cases. The modified ceramics and multi-surface hollow balls with biofilms were observed by environmental scanning electron microscope during the stable operational phase (ESEM XL-12) in the Testing Center of Yangzhou University in China. The microstructures of the multi-surface hollow balls without (Supplementary Fig. 2a), and with, biofilms (Supplementary Fig. 2b) showed that the multi-surface hollow balls had suitable particle size, a solid structure, and a smooth dense surface with rare microspores and spiny-warty projections. Thus the multi-surface hollow balls, which are not conducive to microbial attachment, were effective in increasing the spacing between the packing materials, increasing the gas–liquid mass transfer efficiency, and reducing the drop in pressure between packing grains. The modified ceramics had a suitable particle size, solid structure, and an uneven and rough surface with evenly covered small ceramic particles of different sizes and shapes with high porosity (Supplementary Fig. 2c). The modified ceramics had a high total area and specific surface area, and were conducive to microbial attachment. We confirmed that there were a large number of microorganisms on the surfaces of the modified ceramics, and in the spaces between the small ceramic particles, during the stable operational phase of the BTF (Supplementary Fig. 2d). (TIFF 888 kb)

CB levels as affected by *v.* **a.** Chlorobenzene **(**CB) gas inlet (*C*_in_) and outlet (*C*_out_) concentration and inlet loading rate (*ILR*) at different spray liquid flow rates. **b.** CB removal efficiency (*RE*) and elimination capacity (*EC*) at different spray liquid flow rates. An inappropriate *v* could lead to failure of the BTF. To test this, *C*_in_ was maintained at around 1200 mg m^-3^ and 0.4 m^3^ h^-1^ for *Q*, giving an EBRT of 56 s. While other parameters remained unchanged, *C*_in_ and *C*_out_ could be detected as *v* was changed after each replacement of the spray fluid. The results showed that the *C*_in_ and *ILR* in the BTF remained unchanged. When *v* was increased, *C*_out_ first decreased to a value of 88.79 mg m^-3^, and then increased again (Supplementary Fig. 3a). Correspondingly, *EC* and *RE* increased to a maximum and then decreased, although both these changes were small (Supplementary Fig. 3b). These results showed that *v* does not significantly affect the CB levels, while a small effect is seen on *C*_out_. When *v* was about 27.6 ml min^-1^, *C*_out_ reached a minimum that met the integrated emission standard of air pollutants of China (CAIES). At the same time, *EC* and *RE* were maximized. Considering the negative effects of increasing *v*, *v* should be controlled at about 27.6 ml min^-1^. (TIFF 180 kb)

## Abbreviations

BTF: Biotrickling filter
CB: Chlorobenzene
*C*_in_: The CB gas inlet concentration
*C*_out_: The CB gas outlet concentration
EBRT: The empty bed retention time
*EC*: The CB elimination capacity
*ILR*: The CB gas inlet loading rate
*Q*: The CB gas inlet flow rate
*RE*: The CB removal efficiency
VOCs: Volatile organic compounds
*v*: The spray liquid flow rate
