# Supplementary figures and images for: The operating performance of a biotrickling filter with Lysinibacillus fusiformis for the removal of high-loading gaseous chlorobenzene
Source: Biotechnol Lett. 2014 Jun 15;36(10):1971–9. doi: 10.1007/s10529-014-1559-5 (PMC4150996; doi:10.1007/s10529-014-1559-5)

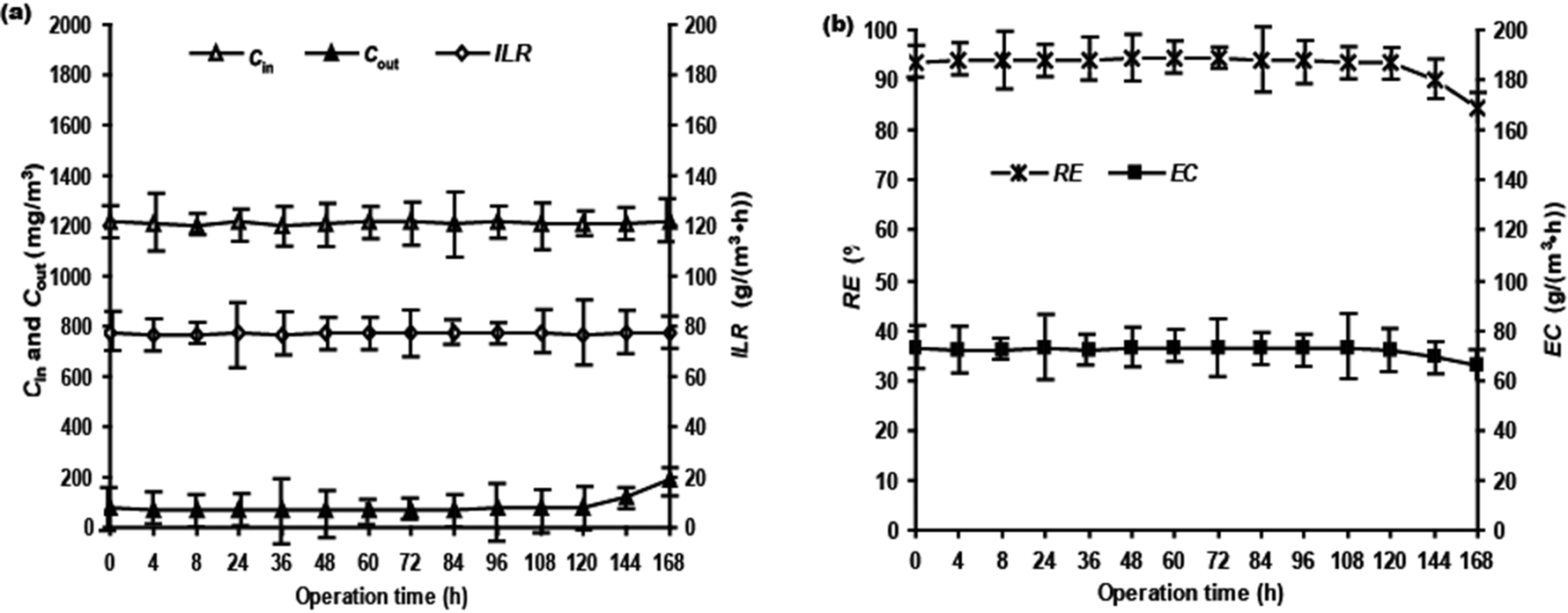

Supplement: Supplementary file 1 — Chlorobenzene (CB) level within a cycle period. a CB gas inlet (Cin) and outlet (Cout) concentrations and inlet loading rate (ILR) within a cycle period. b CB removal efficiency and CB elimination capacity within a cycle period. Over one cycle of operating the BTF, the CB gas inlet concentration (Cin) and inlet loading rate (ILR) were stable and fluctuated within the appropriate range. However, the CB gas outlet concentration (Cout) initialy maintained an acceptable fluctuation and then gradually increased after 96 h and exceeded the integrated emission standard of air pollutants of China (CAIES) at 144 h Supplementary Fig. 1 (Part a). Nevertheless, the CB removal efficiency (RE) and the CB elimination capacity (EC) held steady in the appropriate range although they fluctuated significantly until 144 h when they rapidly decreased. Supplementary Fig. 1 (Part b) shows that the increase in Cout was the reason behind the reduction in RE and EC. Therefore, Cout was the most direct and sensitive indicator of CB purifying performance in the BTF. The CB purifying effect decreased after extended operation of the BTF for 6-7 d under a constant spray liquid. [file 10529_2014_1559_MOESM1_ESM.tif]

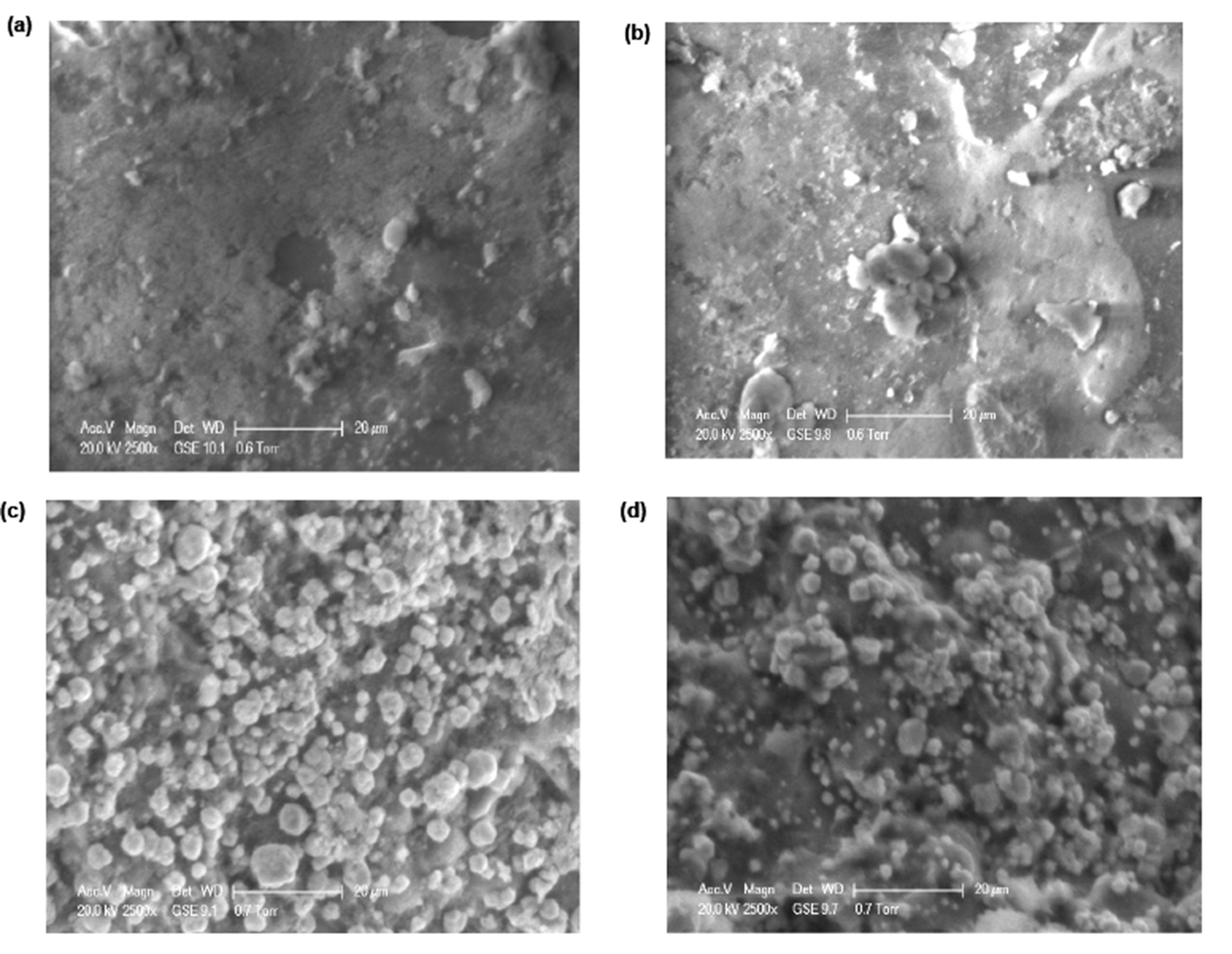

Supplement: Supplementary file 2 — Microstructure analysis of the packing materials with biofilms by ESEM. a ESEM of a multi-surface hollow ball with no biofilms. b ESEM of a multi-surface hollow ball with biofilms (× 2500). c ESEM of modified ceramics with no biofilms. d ESEM of modified ceramics with biofilms. Scale marker bars = 20 µm in all cases. The modified ceramics and multi-surface hollow balls with biofilms were observed by environmental scanning electron microscope during the stable operational phase (ESEM XL-12) in the Testing Center of Yangzhou University in China. The microstructures of the multi-surface hollow balls without (Supplementary Fig. 2a), and with, biofilms (Supplementary Fig. 2b) showed that the multi-surface hollow balls had suitable particle size, a solid structure, and a smooth dense surface with rare microspores and spiny-warty projections. Thus the multi-surface hollow balls, which are not conducive to microbial attachment, were effective in increasing the spacing between the packing materials, increasing the gas–liquid mass transfer efficiency, and reducing the drop in pressure between packing grains. The modified ceramics had a suitable particle size, solid structure, and an uneven and rough surface with evenly covered small ceramic particles of different sizes and shapes with high porosity (Supplementary Fig. 2c). The modified ceramics had a high total area and specific surface area, and were conducive to microbial attachment. We confirmed that there were a large number of microorganisms on the surfaces of the modified ceramics, and in the spaces between the small ceramic particles, during the stable operational phase of the BTF (Supplementary Fig. 2d). (TIFF 888 kb) [file 10529_2014_1559_MOESM2_ESM.tif]

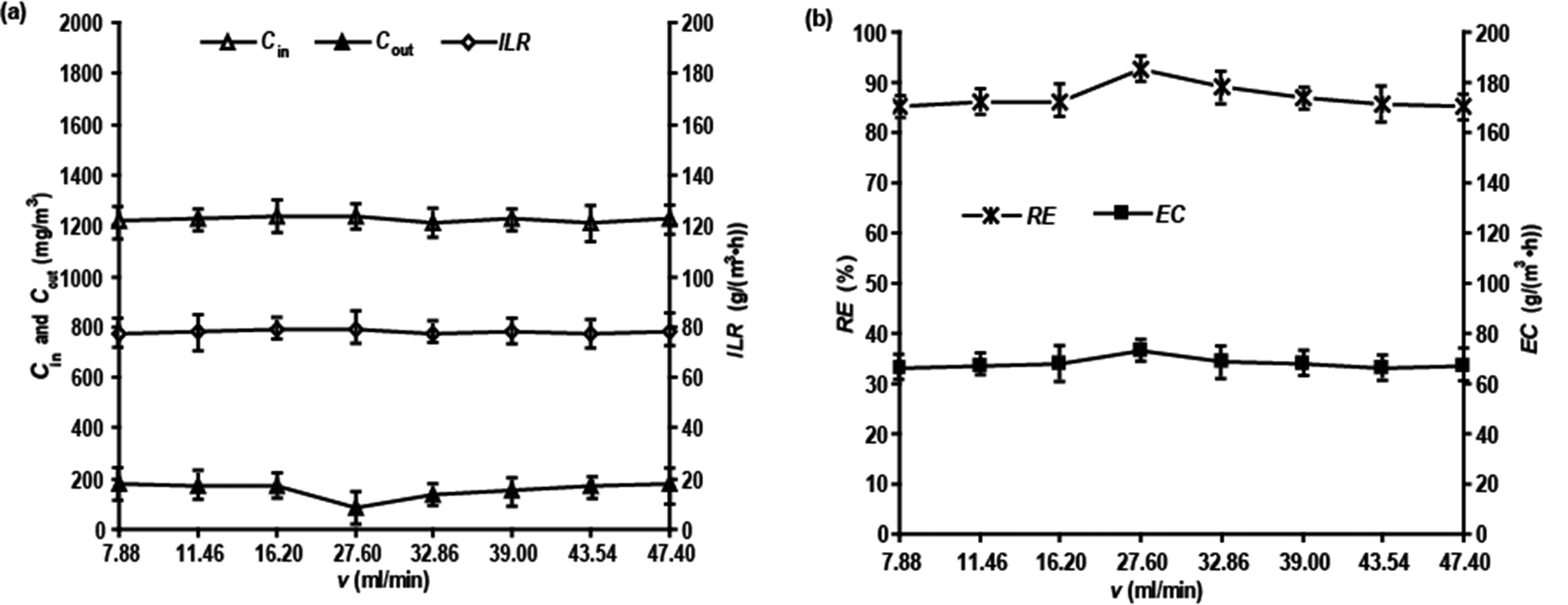

Supplement: Supplementary file 3 — CB levels as affected by v. a. Chlorobenzene (CB) gas inlet (C in) and outlet (C out) concentration and inlet loading rate (ILR) at different spray liquid flow rates. b. CB removal efficiency (RE) and elimination capacity (EC) at different spray liquid flow rates. An inappropriate v could lead to failure of the BTF. To test this, C in was maintained at around 1200 mg m-3 and 0.4 m3 h-1 for Q, giving an EBRT of 56 s. While other parameters remained unchanged, C in and C out could be detected as v was changed after each replacement of the spray fluid. The results showed that the C in and ILR in the BTF remained unchanged. When v was increased, C out first decreased to a value of 88.79 mg m-3, and then increased again (Supplementary Fig. 3a). Correspondingly, EC and RE increased to a maximum and then decreased, although both these changes were small (Supplementary Fig. 3b). These results showed that v does not significantly affect the CB levels, while a small effect is seen on C out. When v was about 27.6 ml min-1, C out reached a minimum that met the integrated emission standard of air pollutants of China (CAIES). At the same time, EC and RE were maximized. Considering the negative effects of increasing v, v should be controlled at about 27.6 ml min-1. (TIFF 180 kb) [file 10529_2014_1559_MOESM3_ESM.tif]
